# Evaluation Framework of Large Language Models in Medical Documentation: Development and Usability Study

**DOI:** 10.2196/58329

**Published:** 2024-11-20

**Authors:** Junhyuk Seo, Dasol Choi, Taerim Kim, Won Chul Cha, Minha Kim, Haanju Yoo, Namkee Oh, YongJin Yi, Kye Hwa Lee, Edward Choi

**Affiliations:** 1 Department of Digital Health Samsung Advanced Institute of Health Sciences and Technology (SAIHST) Sungkyunkwan University Seoul Republic of Korea; 2 Department of Nursing Samsung Medical Center Sungkyunkwan University School of Medicine Seoul Republic of Korea; 3 Department of Emergency Medicine Samsung Medical Center Sungkyunkwan University School of Medicine Seoul Republic of Korea; 4 NAVER Digital Healthcare Lab Seongnam Republic of Korea; 5 Department of Surgery Samsung Medical Center Sungkyunkwan University School of Medicine Seoul Republic of Korea; 6 Department of Internal Medicine College of Medicine Dankook University Cheonan Republic of Korea; 7 Department of Information Medicine Asan Medical Center and University of Ulsan College of Medicine Seoul Republic of Korea; 8 Korea Advanced Institute of Science and Technology Daejeon Republic of Korea

**Keywords:** large language models, health care documentation, clinical evaluation, emergency department, artificial intelligence, medical record accuracy

## Abstract

**Background:**

The advancement of large language models (LLMs) offers significant opportunities for health care, particularly in the generation of medical documentation. However, challenges related to ensuring the accuracy and reliability of LLM outputs, coupled with the absence of established quality standards, have raised concerns about their clinical application.

**Objective:**

This study aimed to develop and validate an evaluation framework for assessing the accuracy and clinical applicability of LLM-generated emergency department (ED) records, aiming to enhance artificial intelligence integration in health care documentation.

**Methods:**

We organized the Healthcare Prompt-a-thon, a competitive event designed to explore the capabilities of LLMs in generating accurate medical records. The event involved 52 participants who generated 33 initial ED records using HyperCLOVA X, a Korean-specialized LLM. We applied a dual evaluation approach. First, clinical evaluation: 4 medical professionals evaluated the records using a 5-point Likert scale across 5 criteria—appropriateness, accuracy, structure/format, conciseness, and clinical validity. Second, quantitative evaluation: We developed a framework to categorize and count errors in the LLM outputs, identifying 7 key error types. Statistical methods, including Pearson correlation and intraclass correlation coefficients (ICC), were used to assess consistency and agreement among evaluators.

**Results:**

The clinical evaluation demonstrated strong interrater reliability, with ICC values ranging from 0.653 to 0.887 (*P*<.001), and a test-retest reliability Pearson correlation coefficient of 0.776 (*P*<.001). Quantitative analysis revealed that invalid generation errors were the most common, constituting 35.38% of total errors, while structural malformation errors had the most significant negative impact on the clinical evaluation score (Pearson *r*=–0.654; *P*<.001). A strong negative correlation was found between the number of quantitative errors and clinical evaluation scores (Pearson *r*=–0.633; *P*<.001), indicating that higher error rates corresponded to lower clinical acceptability.

**Conclusions:**

Our research provides robust support for the reliability and clinical acceptability of the proposed evaluation framework. It underscores the framework’s potential to mitigate clinical burdens and foster the responsible integration of artificial intelligence technologies in health care, suggesting a promising direction for future research and practical applications in the field.

## Introduction

Large language models (LLMs) have experienced rapid advancement in recent years, significantly influencing various domains, including health care. This remarkable development is supported by their increasingly sophisticated capabilities in understanding and generating human-like text [1], as seen in models like GPT-4 and its successors [2]. From creative writing and complex problem-solving to multilingual translation and personalized communication, LLMs are reshaping the landscape of numerous industries, transcending the traditional boundaries of the capabilities of artificial intelligence (AI).

Especially, LLMs have shown remarkable capabilities in the health care sector, beginning with their impressive grasp of medical knowledge. This is highlighted by their successful performance on challenging exams, such as the USMLE (United States Medical Licensing Examination), demonstrating their proficiency in medical concepts [3,4]. Moving beyond theoretical knowledge, LLMs are now being applied to practical tasks, including the creation of detailed medical documents, which indicates their potential for direct applications in clinical settings [5-7]. The significant potential of LLMs is further underscored by their applications, which range from augmenting clinical decision-making to providing personalized patient care [8]. Their ability to process vast amounts of medical literature and patient data suggests a transformative impact on both medical research and practice [9,10]. Moreover, the continuous improvement in the performance and accuracy of these models points to a promising future in enhancing health care delivery and patient outcomes.

However, despite these advancements, the integration of LLMs into clinical practice faces significant challenges. Concerns regarding the accuracy of generated content, particularly in scenarios involving hallucinations or fabrications [6,11,12], pose substantial risks in the high-stakes environment of health care. This issue goes beyond just the limitations of the LLM models themselves; it is significantly compounded by the absence of objective and reliable standards for evaluating the generated content. The lack of clear criteria on what constitutes good output or an effective prompt and the direction for necessary improvements lead to uncertainty in their practical application. In medical settings, where precise and accurate information is vital, this absence of clear standards for prompt efficacy and output evaluation poses significant hurdles to their practical implementation, hindering the reliable and safe integration of LLMs into clinical practices.

Our research focuses on 2 primary goals. First, we aim to establish a framework for evaluating the outputs of LLMs in medical records. This includes identifying and quantifying errors and benchmarking these against expert opinions. Second, our study seeks to assess whether the outcomes produced by LLMs are clinically acceptable and reliable, examining their potential to support clinical decision-making processes. By achieving these objectives, we strive to enable the safe and effective integration of LLMs into health care, thereby enhancing patient care and clinical operations.

## Methods

### Data Collection Process

#### Overview

To evaluate the potential of LLMs in health care, particularly in generating patient records, we organized the Healthcare Prompt-a-thon. This event was specifically designed to gather prompts and their outputs from participants interested in health care–focused generative LLMs. In this section, we discuss the process of the prompt collection, encompassing its organization, task creation, implementation of the competition, and the application of LLM technology. This comprehensive approach ensures a thorough understanding of how the event was structured and executed, as well as how the LLM technology was applied in this context.

#### Overall Information of the Healthcare Prompt-a-Thon

The Healthcare Prompt-a-thon, organized by the Korea Society of Artificial Intelligence in Medicine under the generative model research group and hosted by Samsung Medical Center with sponsorship from NAVER Cloud, aimed to explore the potential of LLMs in health care. The event registration, which was open from October 20 to November 17, 2023, through Social Network Services and internal hospital web networks, attracted participants interested in the application of generative models in health care documentation.

**Figure 1:**
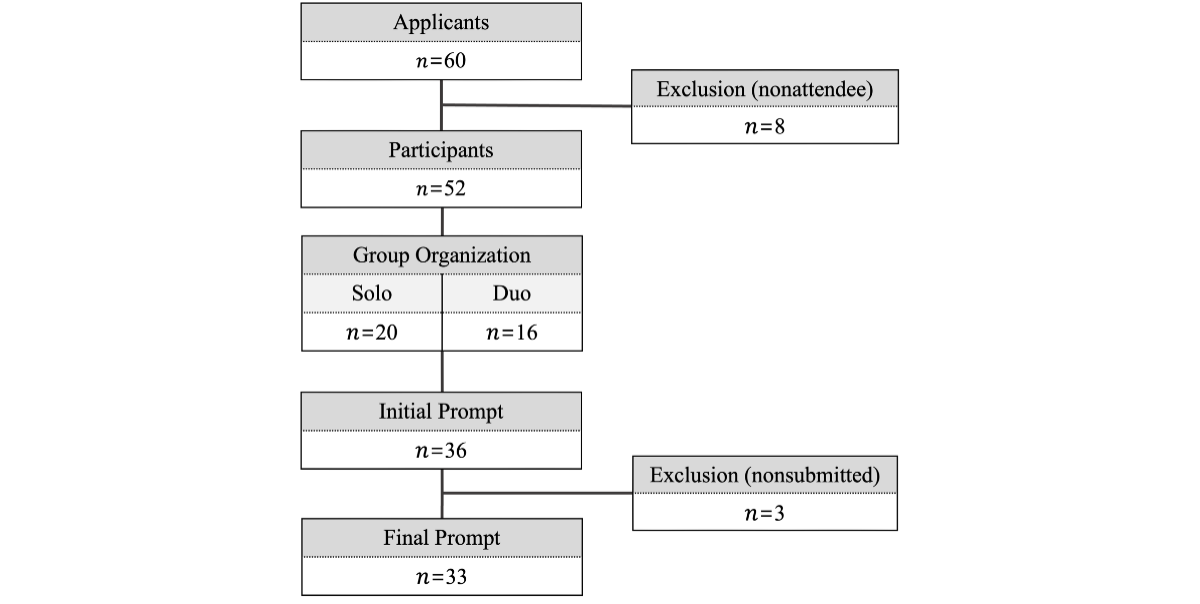
Prompt collection flow.

On November 17, 2023, at 115 Ilwon-ro, Gangnam-gu, Seoul, a total of 52 participants (comprising 20 solo and 16 duo teams) attended the event. These teams, made up of both solo and duo participants, contributed 33 prompts for evaluation. The prompt collection flow of the event is depicted in Figure 1.

The majority of participants were from health care organizations, medical centers, and universities, with a significant representation of researchers, master’s students, and doctors, including radiologists. This diverse demographic underscored the health care focus and interdisciplinary nature of the event.

#### Task Development

The development of the tasks for the Healthcare Prompt-a-thon involved a collaborative effort spanning 2 months, engaging a diverse team of 5 professionals including an emergency medicine physician, transplant surgery physician, critical care nurse, engineer, and linguist. This team diligently created a new dataset, structuring it according to standard hospital dataset formats to ensure its relevance and applicability in a clinical setting. Although the data were simulated, they were carefully designed to mirror plausible scenarios in health care, leveraging the extensive expertise of the medical professionals on the team.

**Figure 2:**
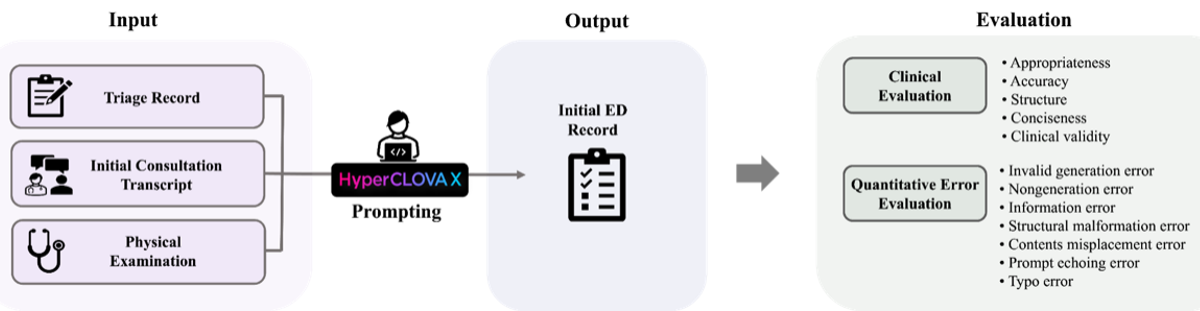
Process flow for generating initial emergency department (ED) records. The flow begins with inputting patient data (triage record, consultation transcript, and physical examination), followed by a “prompting” phase to create the initial ED record. The output is evaluated through “clinical evaluation” and “quantitative evaluation” for comprehensive assessment.

The competition comprised 3 main categories of tasks: creating initial emergency department (ED) records, comparing prescriptions, and generating discharge records. However, the primary focus of our study, as well as the evaluation process, was centered on the creation of initial ED records. For this main task, participants used detailed case scenarios provided to them to develop prompts. These prompts were designed to generate initial ED records based on various inputs, including prescreening notes, transcripts of dialogues between doctors and patients, and physical examination data. The process and nature of these tasks are illustrated in Figure 2.

To guide the participants, 2 example patient cases were provided, complete with input datasets and ideal responses for an initial consultation. These examples served as benchmarks for the participants, aiding in the development of their prompts. The evaluation of these prompts was carried out using a specially curated, unreleased patient dataset, enabling an objective assessment of their effectiveness in simulating real-world medical scenarios. The complexity and organization of these cases went beyond typical medical records, making them exemplary for both input and output in the competition. More detailed information about the patient cases can be found in Multimedia Appendix 1.

#### Competition Process

The Healthcare Prompt-a-thon was structured to maximize participant engagement and creativity. One week prior to the event, participants received a set of practice problems and descriptions of medical records to familiarize themselves with the tasks. On the day of the event, we unveiled the actual examples to be used in the competition.

At the outset of the event, we provided detailed explanations of the 3 main tasks: creating initial ED records, comparing prescriptions, and generating discharge records. Following this, participants were introduced to the LLM platform, along with a comprehensive tutorial on its use and strategies for effective prompt creation. This training was crucial in equipping participants with the necessary skills to navigate the LLM platform and create high-quality prompts.

The task execution phase spanned approximately 5 hours, from 10:00 AM to 3:00 PM, during which participants worked on generating the required outputs. Following the completion of the event, participants submitted their prompts through a Google Form. Each participant’s prompts were submitted once to the LLM for evaluation. This approach was chosen to streamline the competition process and ensure consistency in the evaluation of outputs. These prompts were then executed in real time, and the generated results were used as the basis for evaluation. The evaluation of these prompts was conducted in the subsequent 1.5 hours. The judging panel, consisting of experts in relevant fields, assessed the submissions based on predefined criteria. The event culminated in an award ceremony, where the top 3 teams were recognized for their outstanding prompts and innovative solutions.

#### Using LLM in Our Process

Among various LLMs available for our study, we selected HyperCLOVA X developed by NAVER Corporation [13]. This decision was informed by HyperCLOVA X’s specialized capabilities in processing the Korean language, an essential requirement for our project’s focus on health care documentation in Korea.

HyperCLOVA X is a Korean-specialized LLM intricately designed to efficiently process and understand the complexities of the Korean language. It builds upon the strengths of its predecessor, CLOVA, with significant enhancements in language processing and generation, having been trained on an extensive database of over 560 billion Korean tokens. These improvements are evident in its outstanding proficiency in understanding and generating Korean text [14]. The integration of HyperCLOVA X with Clova Studio, a user-friendly graphical user interface [15], facilitates intuitive in-context interactions and lowers technical barriers for users, making it an ideal choice for real-time applications in health care settings [16-18].

### Evaluation Methodology

#### Overview

To comprehensively evaluate the initial ED records generated through prompts, we used 2 distinct evaluation methodologies: (1) clinical evaluation by medical experts and (2) quantitative evaluation for objectively assessing errors in the generation process. The development of these evaluation criteria was a collaborative effort involving 3 experts, including an emergency medicine professor, a critical care nurse, and a linguist.

The process of developing the evaluation criteria involved several key steps to ensure their effectiveness and relevance. Initially, we conducted an extensive literature review to gather existing standards and best practices for medical documentation, including guidelines from reputable medical organizations and previous research on AI applications in health care. One notable reference was the Physician Documentation Quality Instrument (PDQI-9), which provides a comprehensive framework for evaluating the quality of medical records [19,20]. However, given the broad scope and time-consuming nature of the PDQI-9, we simplified the criteria to make them more practical for real-time evaluation by our assessors.

Next, the initial framework was presented to the panel of experts, who engaged in several rounds of discussions to refine the criteria. These discussions were crucial for integrating diverse perspectives and ensuring that all critical aspects of medical documentation were covered. Experts emphasized the importance of detailed subcriteria for specific areas such as the presence of hallucinations, missing information, and clinical reasoning. This feedback led to further refinement of the criteria.

To ensure the effectiveness and relevance of our evaluation criteria, a preliminary trial was conducted 2 weeks prior to the main event. This trial involved 10 randomly selected volunteers who engaged in solving prompt problems and generating outputs. The outcomes of this preliminary phase were instrumental in providing practical insights and feedback, which were crucial for refining and enhancing the evaluation criteria to be used in the competition. This process helped ensure that our evaluation criteria were both rigorous and aligned with real-world scenarios, thereby enhancing the validity of our study’s findings.

#### Clinical Evaluation

For the clinical evaluation, we established criteria relevant to assessing the quality of medical record outputs, including appropriateness, accuracy, structure/format, conciseness, and clinical validity. These criteria were meticulously developed to cover key aspects of clinical documentation and are detailed in Table 1. Each element was evaluated using a 5-point Likert scale, where higher scores indicate a stronger presence of the evaluated quality attribute.

**Table 1:** Description of our combined criteria for evaluating prompt outputs: clinical evaluation criteria and quantitative evaluation criteria.

Category			Description
**Clinical** **evaluation** **criteria**			
	Appropriateness	Whether the content of the output corresponds to each item appropriately, ensuring it fits the clinical context rather than just individual correspondence.	
	Accuracy	How well the output matches the provided information, including the presence of hallucinations or missing information.	
	Structure/format	If the output has an appropriate structure and format, with content organized as per the specified criteria rather than just in paragraph form.	
	Conciseness	The length of the output, aiming for brevity without compromising essential information.	
	Clinical validity	If the output exhibits sound clinical reasoning and is deemed acceptable within the context of medical practice.	
**Quantitative** **evaluation** **criteria**			
	Invalid generation error	Instances where the output includes content that should not have been generated, or outputs information not provided in the input data.	
	Nongeneration error	Cases where necessary items are not included in the output, or when the output lacks content that should have been generated.	
	Information error	Occurrences where specific information (such as numerical values, frequency, terminology, and places) is inaccurately generated or represented.	
	Prompt echoing error	Errors in the output that directly reflect the content of the prompt.	
	Structural malformation error	Cases where necessary items are missing or unnecessary items are included in the output.	
	Contents misplacement error	Instances where content that belongs to one category is incorrectly placed in another.	
	Typo error	Errors involving typos or grammatical mistakes in the generated text.	

The evaluation was conducted by a panel of 4 medical experts, consisting of 3 physicians and a surgeon, all of whom are bioinformatics researchers. The evaluations were carried out in 2 phases: a collective assessment during the Healthcare Prompt-a-thon and subsequent independent evaluations to ensure thoroughness and mitigate bias. To minimize potential memory effects and learning bias from the first evaluation phase, a washout period of 1 month was observed before conducting the retest evaluations. Additionally, a consistency analysis was performed to gauge the uniformity of judgments across the panel of medical experts. The outcomes of both the test-retest evaluations and the consistency analysis are elaborated in the results section.

#### Quantitative Evaluation

In addition to clinical evaluation criteria, we introduce a novel quantitative evaluation framework specifically designed for assessing LLM outputs in health care. This framework’s establishment was driven by a thorough review of the generated outputs, during which we identified recurring error patterns and grouped them into distinct categories. Consequently, errors in LLM-generated medical records have been categorized into 7 types: invalid generation error, nongeneration error, information error, prompt echoing error, structural malformation error, contents misplacement error, and typo error. These categories were formed based on the frequency and nature of errors observed in our comprehensive review of the LLM outputs. Detailed definitions of these error types are presented in Table 1.

Furthermore, specific examples of each error type are provided in Table 2. These examples illustrate the practical implications of the identified errors and provide insight into the types of inaccuracies that occurred in the LLM-generated medical records.

To facilitate a structured and detailed evaluation, we categorized the essential components of the initial ED records into 11 distinct items: chief complaint, vital signs, present illness, past history, personal and social history, system inquiry, physical examination, problem list, differential diagnosis, diagnosis plan, and treatment plan. We then counted the presence and frequency of errors in each item.

The results of this quantitative error analysis serve as the foundation for a comprehensive comparison between quantitative and clinical evaluations. This comparison includes both the overall correlation and a detailed analysis of individual evaluation criteria.

By adopting this dual evaluation approach, we gained a comprehensive understanding of the strengths and weaknesses of LLMs in health care from a quantitative standpoint, while also exploring the relationship between clinical and quantitative evaluations.

**Table 2:** Specific examples of each quantitative evaluation error type.

Error type	Ideal output	Error example	Justification of error
Invalid generation error	Personal and social history Travel history: none	Personal and social history Travel history: none Smoking: none Alcohol consumption: none	Erroneously adds information not present in original data such as smoking and alcohol consumption information.
Nongeneration error	Chief complaint Main symptom: abdominal pain Occurrence date: November 16, 2023, 8:11 PM	Chief complaint Main symptom: abdominal pain	Omits essential information such as the occurrence date.
Information error	Vital signs Blood pressure: 125/79 mm Hg Pulse: 98 beats per min Respiration: 18 per min Temperature: 38 °C Oxygen saturation: 98%	Vital signs Blood pressure: 124/78 mm Hg Pulse: 97 beats per min Respiration: 20 per min Temperature: 38.5 °C Oxygen saturation: 99%	Inaccurately represents specific data such as blood pressure, pulse, etc.
Prompt echoing error	Past medical history None	Past medical history: include a structured history of past surgeries and diagnosed conditions. None	Echoes prompt content instead of providing concise, relevant information.
Structural malformation error	Ideal output does not include the “Patient Arrival Information” category.	Patient arrival information Disease classification: disease Means of arrival: taxi Arrival route: home	Includes a category “Patient Arrival Information” that should not exist in the ideal record.
Contents misplacement error	System inquiry Fever: yes	System Inquiry Medication history: none Allergy history: none Surgical history: none Trauma history: none	Content that should be in “Personal and Social History” category is incorrectly included in “System Inquiry.”
Typo error	Problem list Abdominal pain Fever	Problem list Abdominal pang Fever	Contains a typographical error in the word “Abdominal Pain.”

### Ethical Considerations

During the application process for the Healthcare Prompt-a-thon, all participants were informed that their outputs might be used for research purposes and could potentially be made public. Explicit consent was obtained from each participant, ensuring that they were fully aware of the intended use of their data. While personal information was collected solely for the purpose of managing the event, it was not used for research and was securely discarded after the event concluded. No personal identifying information was collected for the research, and all data used in the study were anonymized to protect participants’ privacy and confidentiality. As a token of appreciation for their participation, the top 3 teams were recognized and awarded special prizes (equivalent to US$613, US$110, and US$70). The research was conducted in compliance with ethical standards, and the institutional review board of Samsung Medical Center approved the study protocol (institutional review board 2023-12-018-001).

### Statistical Analysis Methods

Statistical analyses of the evaluation results were conducted using the Python programming language (Python Software Foundation) along with libraries such as *SciPy*, *Pandas*, and *Pingouin*. The specific versions of these programs used in our analysis were Python 3.10, *Pandas* 1.5.3, *SciPy* 1.11.4, and *Pingouin* 0.5.4.

### Clinical Evaluation Methods

For the clinical evaluation, we used a 2-phase approach consisting of an initial assessment during the event followed by a retest evaluation. Our key statistical tool in this phase was the Pearson correlation coefficient. This coefficient measured the consistency of scores between the initial test and the retest, offering a quantifiable measure of reliability in the evaluators’ judgments.

In addition, we applied the intraclass correlation coefficient (ICC), specifically the ICC(3,k) model, to assess the consistency among different experts in the panel. The ICC was instrumental in evaluating the level of agreement among raters, ensuring the robustness of our evaluation criteria across diverse expert opinions [20]. The *F* statistics associated with the ICC further reinforced the reliability of these evaluations.

### Quantitative Evaluation Methods

Quantitative Evaluation Methods involved categorizing and counting various error types, such as invalid generation error, typo error, and structural malformation error, among others. Our approach was to first analyze the frequency of these errors and then assess their impact on the clinical validity of the LLM outputs.

To understand the relationship between error types and clinical evaluation scores, we used the Pearson correlation coefficient. We also calculated *P* values for each error type and category to ascertain the statistical significance of their correlations with clinical evaluation scores, considering a *P* value less than .001 as indicative of a significant correlation.

Additionally, we extended our analysis to encompass different categories of clinical information, such as chief complaint, vital signs, present illness, and so forth. This allowed us to delve deeper into understanding which aspects of the LLM outputs were most pivotal for clinical accuracy and relevance.

## Results

### Result of Clinical Evaluation

#### Overall Statistical Analysis

Table 3 presents the mean and SD for each criterion of the clinical evaluation, offering a more robust summary of central tendency and variability. In the initial test, the highest mean score was noted for structure/format at 3.30 (SD 0.99), indicating a strong organizational aspect in the responses. Conversely, accuracy had the lowest mean score at 2.61, highlighting a critical area for improvement, with an SD of 0.63 suggesting a tighter cluster of responses around the mean.

**Table 3:** Statistical results of clinical evaluation (n=33).

Item and stage		Mean (SD)	Pearson *r*	*P* value
**Appropriateness**			0.613	<.001
	Test	3.03 (0.90)		
	Retest	3.14 (0.66)		
**Accuracy**			0.643	<.001
	Test	2.61 (0.63)		
	Retest	2.88 (0.58)		
**Structure/format**			0.605	<.001
	Test	3.30 (0.99)		
	Retest	3.43 (0.67)		
**Conciseness**			0.684	<.001
	Test	2.96 (0.76)		
	Retest	3.23 (0.62)		
**Clinical** **validity**			0.789	<.001
	Test	2.99 (0.99)		
	Retest	2.89 (0.73)		
**Overall** **score**			0.776	<.001
	Test	14.89 (3.96)		
	Retest	15.57 (2.84)		

Upon retesting, improvements were notable across the board, with structure/format again achieving the highest mean score at 3.43 and showing a decrease in SD to 0.67, reflecting more consistent evaluations. Conciseness also showed significant progress, moving to a mean of 3.23 with a reduced SD of 0.62, indicating a narrower spread of evaluations. The overall score saw an increase from a mean of 14.89 in the initial test to 15.57 in the retest, with the SD narrowing from 3.96 to 2.84, demonstrating a general improvement in the evaluation criteria across participants.

#### Test-Retest Reliability Analysis

**Figure 3:**
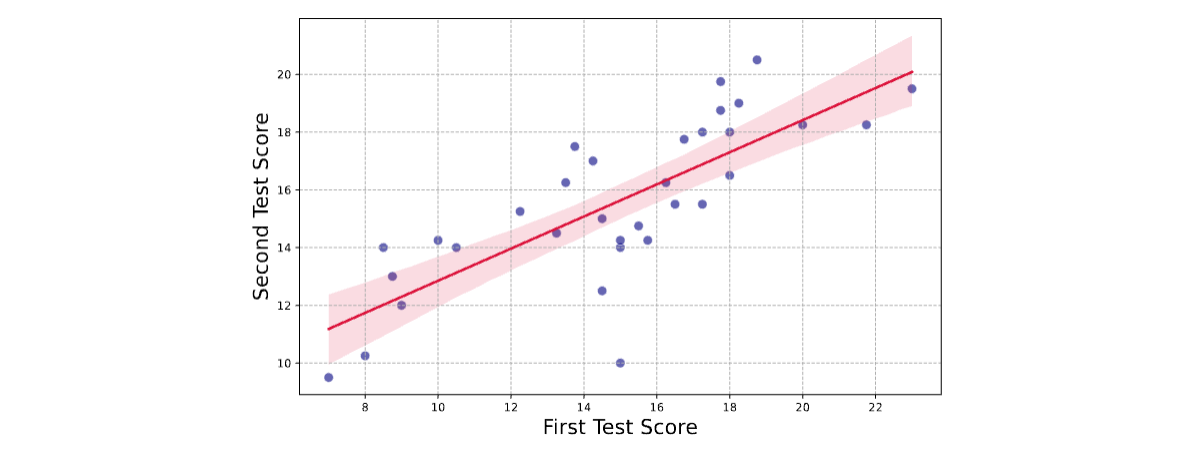
Pearson correlation analysis showing the test-retest reliability of clinical evaluation criteria with a correlation coefficient of 0.776.

Our test-retest reliability analysis [21] method involved 2 distinct testing phases. To quantitatively determine the reliability of our evaluations, we calculated the Pearson correlation coefficient between the average scores of the 2 evaluation sessions. The results, illustrated in Figure 3, show a Pearson correlation coefficient of 0.776, as reported in Table 3. This coefficient indicates a moderate positive correlation, suggesting a reasonable level of agreement among evaluators across both testing sessions. Notably, the clinical validity criterion exhibited the highest correlation, indicating strong consistency in this area, whereas the structure criterion showed a relatively lower correlation, highlighting potential variability in this aspect of the evaluation.

The statistical significance of this correlation was established with a *P* value less than .001, confirming that the observed consistency in the evaluation scores was not due to random chance.

#### Consistency Among Panel

To ensure the robustness of our clinical evaluation criteria, external validation was performed by a diverse panel of domain experts. ICC, particularly using the ICC(3,k) model, provide a measure of the reliability or consistency of measurements made by multiple observers measuring the same quantity [22]. As summarized in Table 4, all items demonstrated strong ICC, when using ICC(3,k) model, with values ranging from 0.653 to 0.887. These ICC values indicate a high level of agreement among raters, with Clinical Validity showing the highest consistency (ICC=0.887). The *F* statistics, ranging from 2.883 to 8.826 for different criteria, further substantiate the reliability of the evaluations, suggesting high agreement consistency among the evaluators. Additionally, the 95% CI for each criterion reinforce the precision of these assessments. Importantly, all ICC values were statistically significant (*P*<.001), underscoring the overall consistency and reliability of the assessment across the various criteria evaluated.

**Table 4:** Intraclass correlation coefficient (ICC) of the clinical evaluation (n=33, ICC3k).

Item	ICC	*F* test (*df*)	*P* value	95% CI
Appropriateness	0.822	5.618 (32, 96)	<.001	0.7-0.9
Accuracy	0.755	4.080 (32, 96)	<.001	0.58-0.87
Structure/Format	0.839	6.203 (32, 96)	<.001	0.73-0.91
Conciseness	0.653	2.883 (32, 96)	<.001	0.41-0.81
Clinical validity	0.887	8.826 (32, 96)	<.001	0.81-0.94
Overall score	0.884	8.609 (32, 96)	<.001	0.8-0.94

### Comparing Clinical and Quantitative Evaluations

#### Quantitative Error Analysis

In the quantitative evaluation of the LLM’s output, we analyzed the frequency of different types of errors. Invalid generation error was the most common error type, constituting approximately 35.38% (75/212) of the total errors. This indicates a significant area where the LLM may need refinement. On the other hand, typo error was found to be the least frequent, accounting for only about 0.47% (1/212) of the errors, suggesting a relatively minor issue in the context of the overall error landscape. Other notable error types included structural malformation error (29.25%, 62/212) and nongeneration error (14.62%, 31/212).

#### Overall Correlation Between Quantitative and Clinical Evaluations

In addition to the detailed error-type analysis, we examined the overall correlation between the comprehensive quantitative evaluation of participant outputs and their clinical evaluation scores. We found a negative correlation of –0.633 (*P*<.001) between the quantitative and clinical evaluations. This indicates that higher error rates in LLM outputs, as identified in the quantitative analysis, correspond to lower clinical evaluation scores, underscoring the importance of accuracy in LLM-generated text for clinical relevance.

#### Detailed Correlation Analysis

Our analysis of the correlation between error types in the LLM output and clinical evaluation scores (referenced in Table 5) highlighted significant differences in their impact on clinical validity. Notably, structural malformation error showed the strongest negative correlation (Pearson *r*=–0.654) among different error types, indicating its substantial effect on clinical assessment. This error type, involving incorrect structure or categorization of content, is crucial for the clinical applicability of LLM outputs. Other error types, such as invalid generation error and nongeneration error, exhibited varying degrees of correlation. In contrast, errors like typo error and prompt echoing error had lower correlations, suggesting a lesser impact on clinical validity.

Additionally, our examination, based on the data in Table 6, revealed that errors in certain categories showed a more pronounced correlation with clinical evaluation scores than others. Notably, categories such as differential diagnosis, diagnosis plan, and treatment plan exhibited strong negative correlations with clinical evaluation scores (Pearson *r* values around –0.698). This suggests that inaccuracies or errors in these critical areas greatly diminish the clinical relevance and reliability of the LLM outputs. In contrast, categories like chief complaint and vital signs exhibited lower negative correlation.

**Table 5:** Correlation between error types and clinical evaluation.

Error type	Count, n	Pearson *r*	*P* value
Invalid generation error	75	0.114	.53
Nongeneration error	31	0.138	.44
Information error	15	–0.182	.31
Prompt echoing error	17	–0.022	.90
Structural malformation error	62	–0.654	<.001
Contents misplacement error	11	0.110	.54
Typo error	1	0.073	.69

**Table 6:** Correlation between category-specific errors and clinical evaluation.

Category	Count, n	Pearson *r*	*P* value
Chief complaint	23	–0.037	.25
Vital signs	6	–0.328	.06
Present illness	19	–0.320	.07
Past history	11	–0.149	.41
Personal and social history	41	0.122	.50
Systems review	49	0.199	.27
Physical examination	19	–0.313	.08
Problem list	11	–0.540	.001
Differential diagnosis	9	–0.698	<.001
Diagnosis plan	9	–0.698	<.001
Treatment plan	9	–0.698	<.001
Additional categories	6	–0.020	.91

## Discussion

### Principal Findings

This study introduces and validates a comprehensive framework for assessing the efficacy of language model applications in emergency medical documentation, effectively bridging the gap between cutting-edge AI technologies and their application in critical health care scenarios. Through this discussion, we explore the implications of our findings, focusing on the insights gained from both clinical and quantitative evaluations of language model outputs. Our analysis underscores the framework’s reliability and clinical pertinence, supported by rigorous analyses that include test-retest reliability, consistency across expert evaluations, and the impact of different types of errors on the utility of the generated documents. Integrating statistical data with expert opinions, we identify the strengths of language models in emergency medical documentation and pinpoint areas needing improvement, emphasizing the framework’s contribution to enhancing AI applications in health care.

### Interpreting the Results of Clinical Evaluation

Upon examining the statistical data from the clinical evaluation, it is notable that the highest median score in both the initial test and upon retesting was seen in structure/format. This indicates that, on average, the LLM outputs were acceptable in terms of organizing and formatting information consistent with the requirements of medical documentation. On the other hand, accuracy presented the lowest median score, pinpointing a significant area for improvement. The lower scores in accuracy imply that the LLM outputs occasionally included errors or inaccuracies in the medical information provided, underscoring the need for further refinement of LLMs to ensure the reliability of generated content.

From the perspective of test-retest analysis, the Pearson correlation coefficient of the overall score confirms that a consistent and reliable evaluation was conducted by the experts, affirming the utility of our framework. Moreover, all 5 criteria—appropriateness, accuracy, structure/format, conciseness, and clinical validity—exhibited correlation coefficients exceeding the *P* value of .60. This demonstrates that our framework is consistent and reliable even across all clinical evaluation criteria. Notably, clinical validity reached the highest correlation coefficient. Given that clinical validation is the most critical element in evaluating medical records, this result signifies that our evaluation is trustworthy and valuable in a clinical context.

Furthermore, the uniformly high ICC results, which measure the agreement level among different evaluators on the same measure, indicate a high level of agreement. Clinical validity, in particular, demonstrated the greatest consistency across the panel. This clarity illustrates that our framework can facilitate consistent clinical evaluations from the perspective of various experts. Appropriateness and structure/format also scored highly, indicating minimal variation in their assessment among the evaluators. These observations, supported by significant *F* statistics and narrow CI, emphasize the assessments’ reliability and precision.

### Comparing Clinical and Quantitative Evaluations

In the process of comparing clinical and quantitative evaluations, a significant relationship was revealed between the number of errors in LLM outputs and their clinical evaluations. This relationship manifested as a negative impact on the overall correlation between quantitative evaluations and clinical scores. This finding suggests that errors identified through quantitative analysis are aligned with clinical assessments, reinforcing the validity of the LLM outputs.

Among the various types of quantitative errors, structural malformation error had the most significant influence on clinical evaluation. This underscores the critical importance of accurate structure and format within medical records, where the presence of unnecessary items or the omission of necessary ones can significantly undermine the document’s utility. Such errors disrupt the logical flow and completeness of medical records, making them less useful in clinical settings. Despite invalid generation error being the most frequently observed type, its impact on clinical evaluation was less pronounced. This could be due to the evaluators’ tendency to overlook certain errors that commonly occur in clinical settings, such as incorrectly labeling unexamined items as “None” rather than “Unknown.”

Further analysis of category-specific errors revealed that specific categories within the initial ED records, such as differential diagnosis, diagnosis plan, and treatment plan, were most critical to clinical evaluators. These categories, pivotal for clinical decision-making, demonstrated a strong correlation with clinical evaluations, underscoring the significant impact of inaccuracies in these critical reasoning areas on clinical relevance. This reflects the inherent value clinicians place on deep clinical reasoning within medical records.

Conversely, categories like system inquiry and personal and social history, despite being areas where errors were frequently found, showed lesser impact on clinical evaluation. This may be attributed to the prevalence of similar errors in human-generated records, leading to a more forgiving attitude from evaluators. Notably, instances of mislabeling unexamined items as “None”—a type of invalid generation error previously discussed—within these categories were often overlooked.

This nuanced analysis highlights the differential weighting of error types by expert evaluators, demonstrating that while quantitative error analysis is essential for identifying and categorizing errors, the subjective judgment of medical professionals remains indispensable. The evaluators’ prioritization underscores the critical role of clinical reasoning in assessing the relevance and acceptability of LLM outputs, illustrating the complex interplay between objective error identification and subjective clinical judgment in determining the utility of AI-generated medical documentation.

### Integrating Automated Quantitative Assessments With Clinical Expertise

One area for future development involves integrating automated quantitative evaluations with the nuanced understanding of clinicians. Our study highlights the importance of balancing objective error analysis with subjective clinical judgment. Future research could explore creating systems that automate the quantitative evaluation process while incorporating clinician input to refine these assessments. This integration would aim to harness the strengths of both AI and human expertise, ensuring more accurate and clinically relevant LLM outputs in health care settings.

Future development should focus on integrating automated quantitative evaluations with clinicians’ nuanced understanding. Automated systems, such as machine learning algorithms, could classify error types and correlate these with clinical expert assessments. This would balance the objective error analysis with subjective clinical judgment, reducing cognitive load on clinicians while ensuring the accuracy and clinical relevance of LLM outputs. Further research could explore refining these systems to improve the applicability of LLM-generated documents in health care settings, thereby enhancing their effective integration into real-world medical documentation.

### Limitations

Our study, while providing significant insights into the application of LLMs in health care, faced certain limitations. The lack of detailed personal data, such as age and gender, from participant profiles limits our understanding of the sample’s diversity and the generalizability of our findings. Additionally, the exclusive use of HyperCLOVA X for generating and evaluating prompts restricts our insights to a single LLM. This presents a limitation in understanding how different LLMs might perform in similar tasks, which is an important aspect for future explorations.

Furthermore, we did not investigate the reproducibility of LLM outputs. The absence of tests to determine whether repeated applications of the same prompts would yield consistent results under varied conditions leaves a gap in understanding the reliability of LLM-generated content in clinical settings. Additionally, our study’s focus on initial records from the ED narrows its scope. Future studies should broaden the research to include a wider range of medical records and clinical departments.

Finally, while the PDQI-9 could have provided a more comprehensive evaluation, we simplified our criteria due to time constraints and the cognitive load of the competitive setting. This simplification was necessary for practical use by assessors, but it highlights an area for future enhancement in evaluating LLM applications in health care documentation.

### Conclusion

Our study introduces a novel and robust framework for evaluating LLMs in health care, particularly focusing on initial ED records. During the Healthcare Prompt-a-thon, we used LLMs and combined clinical insights with quantitative analysis. This approach revealed a strong relationship between the prevalence of quantitative errors and the overall quality of clinical outcomes, underscoring the effectiveness and reliability of our framework in assessing clinical utility.

Moreover, the results validate the clinical acceptability of our framework, suggesting that LLMs, when accurately assessed and used, can be integrated into clinical practice. This advancement opens avenues for LLMs in health care, aligning AI innovations with the critical demands of patient safety and care quality. Future research should focus on refining this framework, further enhancing the practicality and adoption of LLMs in health care environments.

## Appendix

Multimedia Appendix 1 Two patient cases provided to the participants.

## Abbreviations

AI: artificial intelligence
ED: emergency department
ICC: intraclass correlation coefficient
LLM: large language model
PDQI-9: Physician Documentation Quality Instrument
USMLE: United States Medical Licensing Examination
