# Supplementary material for: Evaluation Framework of Large Language Models in Medical Documentation: Development and Usability Study
Source: J Med Internet Res. 2024 Nov 20;26:e58329. doi: 10.2196/58329 (PMC11618017; doi:10.2196/58329)
Supplement: Multimedia Appendix 1 [file jmir_v26i1e58329_app1.docx]

# Supplementary

*This script is translated from Korean to English. It may not perfectly capture all the nuances and specific details of the original information.

Case1: Chest pain

# Triage Record

■ Patient Basic Information

- Gender/Age: Male/53

- Arrival Date and Time: 2023-11-17 21:00

■ Initial Patient Evaluation

- Onset Time: 30 minutes ago

- Blood Pressure: 150/90 mmHg

- Pulse: 110 beats/min

- Respiration: 22 breaths/min

- Temperature: 36.6°C

- Oxygen Saturation: 98%

- Travel History: None

- Initial KTAS: Level 2

- Patient Response: Alert

- Pain Assessment: Yes

- Pain Score: 8

- Fall Assessment: 35

■ Visit Information

- Disease Classification: Disease

- Mode of Arrival: 119 (Emergency Services)

- Arrival Route: Home

■ Medical Information

- Department: Emergency Medicine

- Diagnosis: Chest Pain

## Consultation Transcript

Doctor: Hello. What brings you here today?

Patient: I suddenly started experiencing severe chest pain about 30 minutes ago and it's hard to breathe.

Doctor: Can you describe the pain? Does it feel like pressure?

Patient: Uh.. Uh.. It feels like a tight, squeezing pain in the center of my chest. Very strong, and pressing.

Doctor: Does the pain radiate anywhere? For example, to your arms, neck, jaw, or back?

Patient: Yes... Yes... It seems to spread to my left arm and shoulder.

Doctor: What were you doing when the pain started?

Patient: I was just watching TV at home... Ugh...

Doctor: Have you had any heart conditions before? Or do you have a history of chronic diseases like hypertension, diabetes, or high cholesterol?

Patient: No heart disease, just hypertension. My father and uncle died of heart disease.

Doctor: What medications are you currently taking? Do you smoke or drink?

Patient: I take blood pressure medication every day. I drink occasionally, but I don't smoke.

Doctor: Okay. Are you feeling dizzy, nauseous, or have you vomited? Have you experienced sudden cold sweats or clammy skin?

Patient: Yes, I'm dizzy and have cold sweats right now.

Doctor: Okay, let me examine your condition.

# Physical Examination

- Heart Sounds

  - Auscultation: Rapid and irregular

- Lungs

  - Auscultation: Normal

- Abdomen

  - Upper Abdomen: No tenderness or rebound tenderness. Soft and flat.

  - Auscultation: Normal bowel sounds

Case2: Fever

## Triage Record

■ Patient Basic Information

- Gender/Age: Female/43

- Arrival Date and Time: 2023-11-17 21:00

■ Initial Patient Evaluation

- Onset Time: 4 days ago

- Blood Pressure: 125/79 mmHg

- Pulse: 98 beats/min

- Respiration: 18 breaths/min

- Temperature: 39°C

- Oxygen Saturation: 98%

- Travel History: None

- Initial KTAS: Level 3

- Patient Response: Alert

- Pain Assessment: No

- Pain Score: 0

- Fall Assessment: 25

■ Visit Information

- Disease Classification: Disease

- Mode of Arrival: Taxi

- Arrival Route: Home

■ Medical Information

- Department: Emergency Medicine

- Diagnosis: Fever

## Consultation Transcript

Doctor: Hello, what is your name?

Patient: Hello, my name is Na Gureum.

Doctor: What brings you here today?

Patient: I have a high fever and a lot of coughing, so I came from home.

Doctor: When did the fever start?

Patient: Four days ago.

Doctor: Is the fever constant, or does it come and go?

Patient: It comes and goes. It drops a bit when I take antipyretics.

Doctor: Do you feel chills, shivers, or cold sweats?

Patient: I feel chills all over my body, and I shiver. My whole body aches.

Doctor: Do you have a runny nose or cough?

Patient: Yes, I cough all day and have a bit of a runny nose.

Doctor: What about phlegm?

Patient: There's more than usual, it's opaque and white, and it's sticky.

Doctor: Since when did this start?

Patient: Since today, I've had more phlegm and a high fever. My throat is very sore. I've been taking Tylenol every 8 hours, but the fever returns when the effect wears off.

Doctor: Are you experiencing any shortness of breath?

Patient: I'm not sure.

Doctor: Does it hurt when you breathe?

Patient: Yes, it feels a bit uncomfortable when I breathe.

Doctor: Do you wheeze when you breathe?

Patient: No, but I can't taste well. My throat has never hurt this much, it feels like it's tearing apart.

Doctor: Is there anyone in your family or close contacts with cold symptoms?

Patient: A colleague at work recently got COVID-19.

Doctor: Any rashes on your skin?

Patient: No, my skin is fine.

Doctor: Do you have a headache or stiffness in the back of your neck, or does your head throb when you walk?

Patient: I have a slight headache.

Doctor: Any diarrhea or vomiting?

Patient: No, none of that.

Doctor: Do you usually have any respiratory diseases?

Patient: Respiratory diseases? No, I don't think so.

Doctor: Any past illnesses or surgeries?

Patient: I had long-term treatment for varicose veins but no surgery.

Doctor: Any drug allergies or side effects?

Patient: No, none.

Doctor: Okay, let me check your condition.

## Physical Examination

- Facial Pain: None

- Postnasal Drip: None

- Pharyngeal Redness: Present

- Tonsil Enlargement: 1+/1+

- Lymph Node Enlargement: Present

- Auscultation

  - Wheezing: None

  - Crackles: None

- Heart Murmur: None

- Abdomen

  - Bowel Sounds: Normal

  - Tension: None

  - Tenderness: None

  - Rebound Tenderness: None

- Costovertebral Angle Tenderness: Bilaterally absent

- Neck Stiffness: None
